# Dysregulation of TLR2 Serves as a Prognostic Biomarker in Breast Cancer and Predicts Resistance to Endocrine Therapy in the Luminal B Subtype

**DOI:** 10.3389/fonc.2020.00547

**Published:** 2020-04-30

**Authors:** Yunmei Wang, Shuguang Liu, Yanjun Zhang, Jin Yang

**Affiliations:** ^1^Department of Medical Oncology, First Affiliated Hospital of Xi'an Jiaotong University, Xi'an, China; ^2^Shanxi Provincial Cancer Hospital, Taiyuan, China; ^3^Department of Orthopedics, HongHui Hospital, Xi'an, China

**Keywords:** breast cancer, toll-like receptor 2, prognosis, endocrine therapy, resistance, luminal B subtype

## Abstract

**Background:** Breast cancer (BCa) is a serious global health burden among females, and the development of resistance represents an important challenge to BCa treatment. Here, we examined the expression of toll-like receptor 2 (TLR2) in BCa patients and the prognostic value of TLR2 for predicting endocrine resistance.

**Methods:** The study included 150 BCa patients, of which 82 underwent endocrine therapy. TLR2 mRNA expression was measured by quantitative Real-Time PCR, and its prognostic value was determined by Kaplan-Meier survival analysis. Changes in the expression of TLR2 in BCa patients with endocrine resistance were assessed, and the value of TLR2 for predicting endocrine resistance was evaluated using the receiver operating characteristic curve analysis.

**Results:** TLR2 expression was higher in BCa tissue than in normal tissue and associated with tumor size, HER2 status, tumor subtype, and TNM stage. TLR2 upregulation was associated with poor prognosis in patients with BCa, as well as endocrine resistance, and TLR2 upregulation was more prevalent among HER2-positive BCa cases. The predictive performance of TLR2 for endocrine resistance was higher in HER2-positive BCa than in other hormone receptor-positive BCa cases.

**Conclusion:** TLR2 upregulation is a promising biomarker for prognosis and predicting resistance to endocrine therapy. The relationship between TLR2 and HER2 indicates that TLR2 may be involved in endocrine resistance through the HER2 signaling pathway in BCa.

## Introduction

Breast cancer (BCa) is the most frequently diagnosed malignancy and the leading cause of cancer-related mortality among women worldwide (1). There were 2,088,849 new BCa cases and 358,989 deaths from BCa in 2018, accounting for 24.2% of all cancer cases and 15.0% of total deaths among global malignancies in women (2). BCa can be divided into four major subtypes according to the expression status of the estrogen receptor (ER), progesterone receptor (PR), and the human epidermal growth factor receptor 2 (HER2), namely, luminal A, luminal B, HER2+, and triple-negative BCa (3). However, emerging data indicate an increase in the incidence of resistance to endocrine therapy, especially in luminal B BCa (4). Arpino et al. reported that the decreased sensitivity of luminal B BCa to endocrine therapy might be triggered by the crosstalk between ER and HER2 (5). Therefore, novel strategies to inhibit the activity of HER2 may increase the therapeutic efficacy of endocrine therapy in hormone receptor-positive BCa patients.

Toll-like receptors (TLRs) are a family of innate immune receptors that can recognize ligands derived from viruses, bacteria, parasites, and fungi (6). TLRs play important roles in the regulation of inflammatory responses aimed at eliminating pathogen infection and cancer debris (7). In addition, they play regulatory roles in cell proliferation and apoptosis in both normal and tumor cells (8, 9). Increasing evidence supports the double-edged sword effect of TLRs in cancer cells, because uncontrolled TLR signaling leads to a microenvironment that promotes tumor growth (10, 11). The TLR family is divided into two major subtypes according to cellular localization. TLR 1, 2, 5, 6, and 10 are extracellular TLRs, whereas TLR3, 7, 8, and 9 are localized in intracellular organelles (12). TLR2 and TLR4 are two major TLRs that have been widely investigated for their involvement in inflammatory responses and cancers (13). The expression and clinical significance of TLR4 have been reported in patients with BCa (14). TLR2 polymorphisms are associated with BCa risk (10), and TLR2 is involved in immunomodulation in BCa (15). TLR2 is upregulated in most tumors and closely associated with tumor metastasis, playing an important role as a proto-oncogene (16, 17). In colorectal cancer, TLR2 promotes tumor cell proliferation, migration, and invasion by activating the PI3K/AKT signaling pathway (18). However, the clinical significance of TLR2 in BCa remains unclear. Lu et al. showed that TLR2 agonists enhance the efficacy of HER2-targeted monoclonal antibody therapy (19). Considering the important role of HER2 in endocrine therapy resistance in BCa, we investigated the potential role of TLR2 in regulating the sensitivity to endocrine therapy in BCa.

In the current study, changes in the expression of TLR2 in BCa patients and patients with endocrine therapy resistance were investigated, and the clinical significance of TLR2 as a prognostic factor for BCa was evaluated. In addition, we assessed the value of TLR2 for predicting endocrine therapy resistance in patients with luminal BCa.

## Methods and Materials

### Patients and Tissue Samples

The study enrolled 150 patients who were histopathologically diagnosed with BCa in Shaanxi Provincial Cancer Hospital affiliated to the Medical School between 2011 and 2013. None of the patients received anti-tumor therapy before sampling. Of the 150 BCa patients, 68 underwent surgical resection and 82 received endocrine therapy. BCa tissues and adjacent normal tissues were collected from the patients and frozen in liquid nitrogen for further use. The experimental procedures were approved by the Ethics Committee of Shaanxi Provincial Cancer Hospital, and written informed consent was obtained from each participant.

### Clinical Data Collection

The demographic and clinicopathological characteristics of the patients were recorded, including age, tumor size, ER, PR, and HER2 status; tumor subtypes; and TNM stage. ER, PR, and HER2 [FISH (Fluorescence *in situ* hybridization) was used to characterize HER2 expression in IHC (Immunohistochemistry) 2+ cases] status was examined by immunohistochemistry, and BCa subtypes were categorized following the St. Gallen Expert Consensus as follows: luminal A (ER+ and/or PR+, HER2–, Ki-67 <14%), luminal B (ER+ and/or PR+, HER2–, Ki-67 ≥14%; ER+ and/or PR+, HER2+), HER2+ (ER–, PR–, HER2+), and triple-negative (ER–, PR–, HER2–) (20). The TNM stage of BCa patients was determined according to the criteria published by the American Joint Committee on Cancer Classification (21). Overall survival (OS) was defined as the percentage of cases who had been alive after a number of months. The duration is from the beginning of filtering to death. In addition, survival information and the rates of endocrine therapy resistance [primary endocrine resistance was defined as recurrence within 2 years prior to adjuvant endocrine therapy or progression within 6 months prior to first-line endocrine therapy for metastatic BCa (22)] of the patients were collected from a 5-year follow-up survey for the subsequent survival analysis.

### RNA Extraction

Total RNA was extracted from tissues using the TRIzol reagent (Invitrogen, Carlsbad, CA, USA). The concentration and purity of RNA were evaluated using a NanoDrop 2000 (Thermo Fisher Scientific, Waltham, MA, USA). Single stranded cDNA was synthesized from 2 μg RNA using a reverse transcription reagent kit (Invitrogen) according to the manufacturer's instructions.

### Quantitative Real-Time PCR (qRT-PCR)

The mRNA expression of TLR2 in tissue samples was assessed by qRT-PCR using a SYBR Green PCR kit (TaKaRa, Dalian, China) on a 7,500 Real-Time PCR System (Applied Biosystems, USA). GAPDH was used as the internal control gene, and the relative expression of TLR2 was calculated using the 2^−ΔΔCt^ method.

### Statistical Analysis

Data were expressed as the mean ± SD and analyzed using SPSS 18.0 software (SPSS Inc., Chicago, IL, USA) and GraphPad Prism 5.0 software (GraphPad Software, Inc., USA). A K-S test was used to test the normality of TLR2. Differences between groups were analyzed using the Student's *t*-test, one-way ANOVA, or the Chi-square test. Kaplan-Meier (KM) survival curves were generated to determine the prognostic value of TLR2. A receiver operating characteristic curve (ROC) was plotted to assess the value of TLR2 for predicting the occurrence of endocrine resistance. A proportional hazards model (Cox) was used for multi-factor prognostic analysis. A *P* < 0.05 was considered to indicate statistical significance.

## Results

### Expression of TLR2 in Patients With BCa

**Figure 1:**
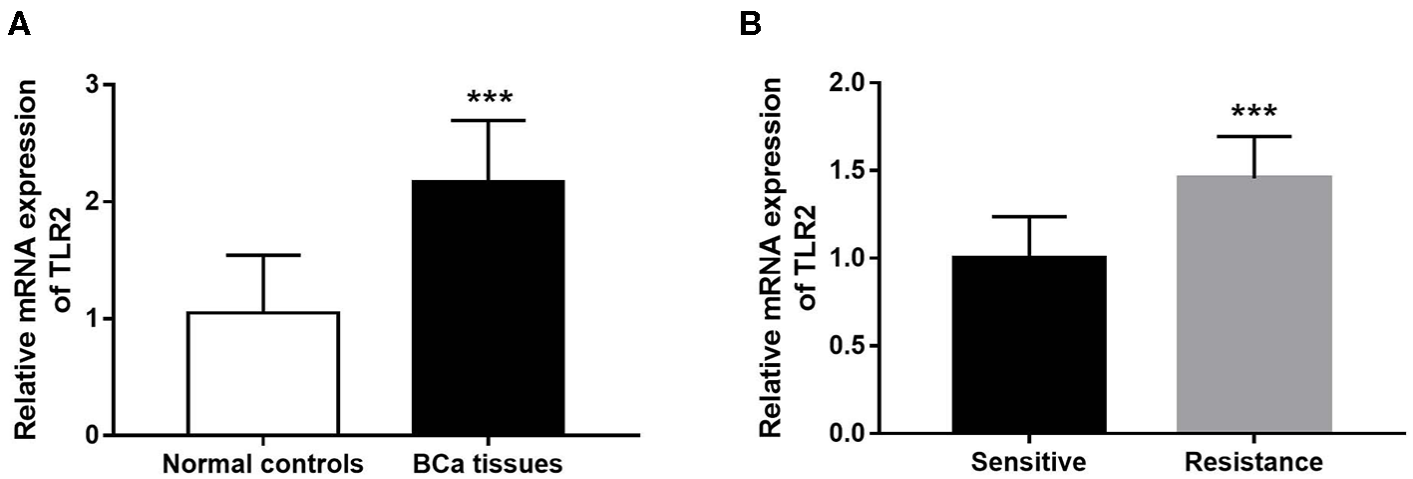
Expression of TLR2 in BCa patients. **(A)** The mRNA expression of TLR2 in BCa tissues was increased compared with the normal controls. **(B)** The relative TLR2 expression was elevated in the BCa patients with resistance of endocrine therapy. ***P < 0.001.

The results of qRT-PCR indicated that TLR2 mRNA expression was significantly higher in BCa tissues than in normal tissues (*P* < 0.001, Figure 1A). In the 5-year follow-up, there were 24 patients with resistance to endocrine therapy, accounting for 29.3% of the 82 BCa patients who received endocrine therapy. TLR2 mRNA expression was significantly higher in the resistant group than in the sensitive group (*P* < 0.001, Figure 1B).

### Association of TLR2 With Clinicopathological Data of BCa Patients

Considering that TLR2 is dysregulated in BCa tissues, this study analyzed the relationship between TLR2 expression and the clinicopathological features of BCa patients. The distribution of TLR2 was a normal distribution (*p* = 0.087). The patients were therefore divided into TLR2- low and -high expression groups according to mean TLR2 expression values. As shown in Table 1, the mRNA expression levels of TLR2 were associated with tumor size, subtypes, and TNM stage (all *P* < 0.05). TLR2 expression was associated with HER2 status (*P* = 0.025). There was no statistically significant association between TLR2 and patient age, ER status, and PR status (all *P* > 0.05).

**Table 1:** Relationship between TLR2 expression and clinicopathological features of BCa patients.

**Features**	**Total No**.	**TLR2 expression**		***P*-values**
	***n* = 150**	**Low (*n* = 72)**	**High (*n* = 78)**	
Age (years)				0.574
≤50	59	30	29	
>50	91	42	49	
Tumor size (cm)				0.036^*^
≤2	70	40	30	
>2	80	32	48	
ER status				0.392
Negative	51	22	29	
Positive	99	50	49	
PR status				0.647
Negative	57	26	31	
Positive	93	46	47	
HER2 status				0.025^*^
Negative	108	58	50	
Positive	42	14	28	
Subtypes				0.001^*^
Luminal A	68	44	24	
Luminal B	42	12	30	
HER2+	18	6	12	
Triple-negative	22	10	12	
TNM stage				0.030^*^
I–II	95	52	43	
III–IV	55	20	35	

ER, estrogen receptor; PR, progesterone receptor; HER2, human epidermal growth factor receptor 2;

* *P < 0.05*.

### Prognostic Value of TLR2 in Patients With BCa

**Figure 2:**
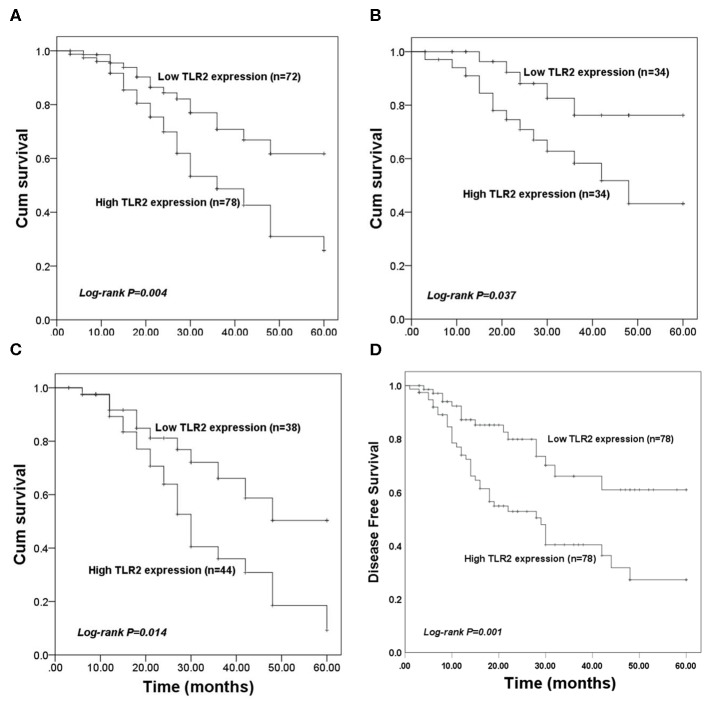
Kaplan-Meier (KM) overall survival curves constructed with the expression of TLR2. **(A)** The KM curves for the total study population. **(B)** The KM curves for patients received surgery. **(C)** The KM curves for patients underwent endocrine therapy. **(D)** The KM disease free survival curves constructed with the expression of TLR2.

The value of TLR2 for predicting the prognosis of BCa was evaluated in the present study. In the entire BCa patient cohort, KM survival curves showed that high TLR2 expression was associated with poor overall survival compared with that of patients with low TLR2 levels (log-rank *P* = 0.004, Figure 2A). In BCa patients who underwent surgery, high TLR2 mRNA levels were also associated with a shorter survival time (log-rank *P* = 0.037, Figure 2B), and a similar pattern was observed in patients receiving endocrine therapy, in which high TLR2 mRNA expression was associated with poor overall survival compared with that of patients with low TLR2 expression (log-rank *P* = 0.014, Figure 2C) While in the entire BCa patient cohort, KM survival curves showed that high TLR2 expression was also associated with poor disease free survival compared with that of patients with low TLR2 levels (log-rank *P* = 0.001, Figure 2D). Moreover, in a Cox model adjusted for method of therapy, TNM stage, level of Her2 and subtypes, patients with high level TLR2 had an elevated hazard ratio, and it was statistically significant (HR 1.87, 95% CI: 1.02–3.40, *p* = 0.042; Table 2). So, the TLR2 level was an independent role to be associated with BCa prognostic.

**Table 2:** Results from a multivariate Cox model for testing the impact of TRL2 on 5-year survival; Method of therapy, TNM stage, level of Her2 and subtypes are adjustment factors.

**Contributing factors**	**N**	**Univariate HR**	**HR**	**95% CI**	***p*-value**
High level of Her2	42	3.103	6.412	1.456–28.236	0.014
High level of TLR2	78	2.3	1.865	1.022–3.406	0.042
Surgery therapy	34	1.809	1.574	0.899–2.757	0.112
TNM stage III–IV	55	3.181	2.168	1.084–4.338	0.029
Luminal A	68				0.039
Luminal B	42	1.245	2.903	1.255–6.713	0.013
HER2+	18	1.31	4.95	1.098–22.222	0.037
Triple-negative	22	4.184	5.495	1.009–30.303	0.049

*95% CI, 95% confidence interval; HR, harzard ratio*.

### The Value of TLR2 for Predicting Endocrine Therapy Resistance

**Figure 3:**
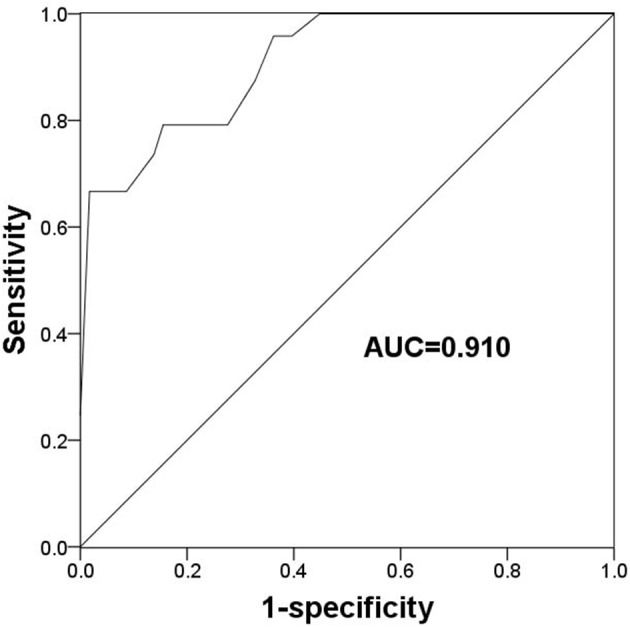
A ROC curve to evaluate the predictive value of TLR2 for the occurrence of endocrine resistance. AUC, area under the curve.

Given the deregulated expression pattern of TLR2 and its association with poor prognosis in patients receiving endocrine therapy, this study used a ROC curve to determine the value of TLR2 for predicting the development of resistance to endocrine therapy which was the cut-off value. As shown Figure 3, the area under the curve (AUC) was 0.910, indicating a high accuracy of TLR2 for distinguishing resistant from sensitive BCa cases. At an optimal cutoff value of 1.228, the sensitivity was 79.2% and the specificity was 86.2%.

### Value of TLR2 for Predicting Endocrine Therapy Resistance in Different Subtypes of BCa

**Figure 4:**
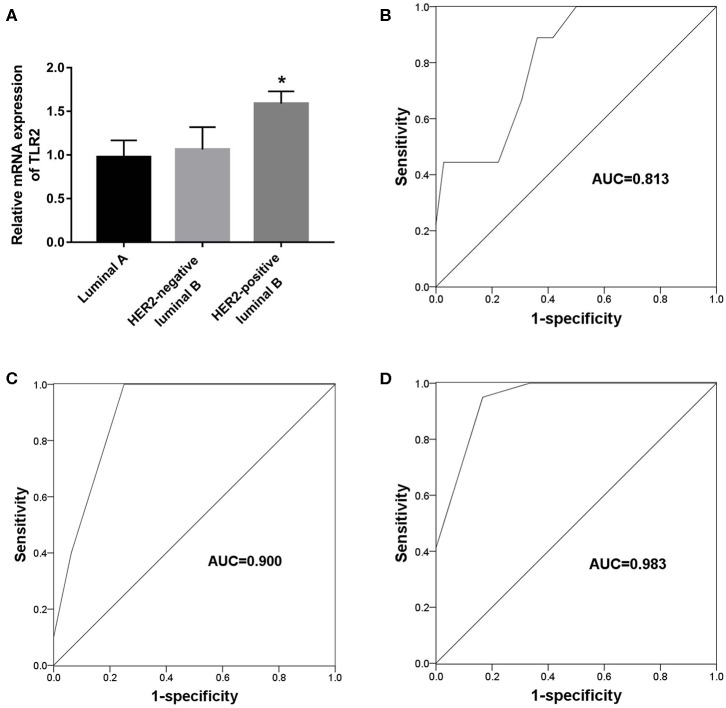
Predictive value of TLR2 for endocrine resistance in different subtypes of BCa patients. **(A)** Expression of TLR2 in different subtypes of BCa patients. **(B)** A ROC curve to evaluate the predictive value of TLR2 for endocrine resistance in luminal A subtype. **(C)** A ROC curve to evaluate the predictive value of TLR2 for endocrine resistance in HER-negative luminal B subtype. **(D)** A ROC curve to evaluate the predictive value of TLR2 for endocrine resistance in HER-positive luminal B subtype. AUC, area under the curve. *p < 0.05 vs Luminal A and HER2-negative luminal B.

The present results suggest an association between TLR2 and HER2 and demonstrate the value of TLR2 for predicting endocrine therapy resistance. Considering HER2 status among BCa subtypes, we further investigated the clinical role of TLR2 in predicting endocrine therapy resistance by dividing BCa patients into luminal A (*n* = 45), HER2-negative luminal B (*n* = 21), and HER-positive luminal B cases (*n* = 16). The resistance rates among BCa patients are listed in Table 3. HER-positive luminal B patients had higher rates of resistance than luminal A and HER2-negative luminal B patients (*P* < 0.01), which was consistent with previous studies reporting the inhibitory effects of HER2 on endocrine therapy sensitivity (23). As shown in Figure 4A, TLR2 mRNA expression was significantly higher in HER2-positive luminal B cases than in luminal A and HER2-negatie luminal B cases (*P* < 0.05). Furthermore, ROC curves based on TLR2 expression indicated that TLR2 could predict the resistance to endocrine therapy in the three subtypes; the AUC for the HER2-positive luminal B cases was relatively high among the three subtypes (AUC = 0.983, Figures 4B–D).

**Table 3:** Endocrine therapy resistance in different BCa subtypes.

**Indicators**	**Luminal A**	**HER2-negative luminal B**	**HER2-positive luminal B**	***P_**1**_***	***P_**2**_***	***P_**3**_***
Total number	45	21	16	0.724	0.002	0.018
Resistance number	9	5	10			
Resistance rate (%)	20.00%	23.80%	62.50%			

*HER2, human epidermal growth factor receptor 2; P1, Luminal A vs. HER2-negative luminal B; P2, Luminal A vs. HER2-positive luminal B; P3, HER2-negative luminal B vs. HER2-positive luminal B*.

## Discussion

BCa is the most common malignancy among women worldwide. This study aimed to provide a novel biomarker for predicting the prognosis of BCa and the resistance to endocrine therapy. The results of the present study indicated that TLR2 mRNA expression was higher in BCa tissues than in normal tissues and associated with tumor size, HER2 status, tumor subtype, and TNM stage. BCa patients with high TLR2 expression had a shorter overall survival than those with low TLR2 expression. In addition, TLR2 was significantly upregulated in patients showing resistance to endocrine therapy, supporting its value as an indicator to predict the development of endocrine resistance in BCa patients. TLR2 expression was higher in the HER2-positive luminal B BCa subtype than in the other hormone receptor-positive BCa cases, and its predictive value for the resistance to endocrine therapy was relatively high in the HER2-positive luminal B subtype.

The incidence and mortality rates of BCa are increasing worldwide (2). Despite advances in BCa treatment methods, the prognosis of patients with this malignancy remains unsatisfactory. Various molecules involved in the initiation and progression of BCa show aberrant expression patterns (24). Qiu et al. found that Cullin 7 is upregulated in BCa tissues and serves as a candidate prognostic biomarker and oncogene (25). Zhang et al. investigated the expression and clinical significance of CD81 in patients with BCa, and provided evidence for CD81 upregulation as a prognostic biomarker and an oncogene that promotes BCa cell migration and proliferation (26). Dai et al. showed that CST1 is upregulated in BCa tissues, and increased CST1 expression predicts a poor prognosis and facilitates tumor progression (27). These studies prompted us to identify novel molecules showing abnormal expression that may serve as biomarkers to improve the prognosis and treatment of BCa.

TLRs play pivotal roles in the activation of immune responses. Studies indicate that TLRs are critical mediators of chronic inflammation in the tumor microenvironment that promote the survival of tumor cells (28). TLR2 is one the most extensively investigated members of the TLR family. West et al. reported that elevated TLR2 level is associated with key genes involved in gastric cancer prognosis and identified TLR2 as a potential therapeutic target in this malignancy (29). Another study in gastric cancer provided additional evidence supporting the role of TLR2 in the inflammatory microenvironment and in promoting tumor progression (30). In glioma, TLR2 facilitates tumor cell immune evasion (31). TLR2 polymorphisms are correlated with the risk of BCa (10) and contribute to BCa cell invasion (32). However, the clinical significance of TLR2 in BCa remains elusive. Analysis of the survival of BCa patients indicated that patients with high TLR2 expression had a poorer prognosis than those with low TLR2 expression. These results indicate that TLR2 upregulation in BCa patients might predict a poor prognosis.

The development of resistance to endocrine therapy, especially in HER2-positive luminal B BCa cases, has attracted increasing attention (33). Dormant/quiescent/slow-cycle cancer stem cells (CSCs) are key factors associated with tumor heterogeneity and are responsible for tumor resistance, migration, and metastatic dormancy, which is the ability of CSCs to survive in target organs and lead to metastasis within 20 years of diagnosis. CSCs interact with the tumor microenvironment to self-renew, resist radiation and chemotherapy, and produce distant metastases (34, 35). BHLHE41 (basic helix-loop-helix family, member e41) and NR2F1 (nuclear receptor subfamily 2 group F member 1) promote the growth of ER positive MCF7 cells *in vivo*. Analysis of patient data suggests that ER-positive diffuse tumor cells with dormancy characteristics are more likely to undergo prolonged dormancy before resuming metastatic growth. (36, 37).

The crosstalk between ER and HER2 may contribute to the resistance to endocrine therapy, as indicated by the enhanced HER2 activity in BCa cases treated with ER inhibitors (5). Identifying novel strategies to improve the therapeutic efficacy of endocrine therapy is critical. Keegan et al. reported that inhibiting PI3K activity may be a strategy to overcome endocrine resistance in BCa (38). Charmsaz et al. demonstrated that the upregulation of S100 β in BCa patients is involved in endocrine resistance and serves as a promising biomarker for the response to endocrine therapy (39). Lee et al. provided evidence supporting the role of MTA1 (metastasis associated 1) as a key regulator of endocrine therapy resistance by modulating autophagy (40). In this study, TLR2 was upregulated in BCa patients with endocrine resistance, and its expression was effective for distinguishing resistant from sensitive cases, indicating the predictive potential of TLR2 for endocrine therapy resistance. In addition, we found an association between TLR2 and HER2. These data combined with the important role of HER2 in endocrine resistance led us to speculate that TLR2 might be involved in the development of endocrine resistance through the HER2 signaling pathway. However, this hypothesis needs to be analyzed and confirmed in further studies.

Taken together, the present data indicate that elevated TLR2 expression is a candidate prognostic biomarker in BCa and may predict the development of endocrine resistance. We identified a relationship between TLR2 and HER2, and showed that the value of TLR2 for predicting the response to endocrine therapy was particularly high in the HER-positive luminal B subtype, indicating that TLR2 may be involved in the regulation of endocrine therapy sensitivity through the HER signaling pathway.

A greater number of samples and validation at different levels may be necessary to verify our results, as this study was limited by a small sample size. In addition, TLR2-related pathways need to be examined in different tissues and cells, and the possible relationship of TLR2 with dormancy in BCa should be investigated in the future.

## Data Availability Statement

The raw data supporting the conclusions of this article will be made available by the authors, without undue reservation, to any qualified researcher.

## Ethics Statement

The studies involving human participants were reviewed and approved by the experimental procedures were approved by the Ethics Committee of shaanxi provincial Cancer Hospital affiliated. The patients/participants provided their written informed consent to participate in this study.

## Author Contributions

YW and SL responsible for collecting data and writing papers. JY and YZ responsible for paper design.

## Conflict of Interest

The authors declare that the research was conducted in the absence of any commercial or financial relationships that could be construed as a potential conflict of interest.

## References

[1] Merino Bonilla JA Torres Tabanera M Ros Mendoza LH. Breast cancer in the 21st century: from early detection to new therapies. Radiologia. (2017) 59:368–79. 10.1016/j.rxeng.2017.08.001 28712528

[2] Bray F Ferlay J Soerjomataram I Siegel RL Torre LA Jemal A. Global cancer statistics 2018: GLOBOCAN estimates of incidence and mortality worldwide for 36 cancers in 185 countries. CA Cancer J Clin. (2018) 68:394–424. 10.3322/caac.21492 30207593

[3] Harbeck N Gnant M. Breast cancer. Lancet. (2017) 389:1134–50. 10.1016/S0140-6736(16)31891-8 27865536

[4] Prat A Baselga J. The role of hormonal therapy in the management of hormonal-receptor-positive breast cancer with co-expression of HER2. Nat Clin Pract Oncol. (2008) 5:531–42. 10.1038/ncponc1179 18607391

[5] Arpino G Wiechmann L Osborne CK Schiff R. Crosstalk between the estrogen receptor and the HER tyrosine kinase receptor family: molecular mechanism and clinical implications for endocrine therapy resistance. Endocr Rev. (2008) 29:217–33. 10.1210/er.2006-0045 18216219 PMC2528847

[6] De Nardo D. Toll-like receptors: activation, signalling and transcriptional modulation. Cytokine. (2015) 74:181–9. 10.1016/j.cyto.2015.02.025 25846205

[7] Chen JQ Szodoray P Zeher M. Toll-like receptor pathways in autoimmune diseases. Clin Rev Allergy Immunol. (2016) 50:1–17. 10.1007/s12016-015-8473-z 25687121

[8] Chung YH Kim D. Enhanced TLR4 expression on colon cancer cells after chemotherapy promotes cell survival and epithelial-mesenchymal transition through phosphorylation of GSK3beta. Anticancer Res. (2016) 36:3383–94. 27354597

[9] Gambara G Desideri M Stoppacciaro A Padula F De Cesaris P Starace D. TLR3 engagement induces IRF-3-dependent apoptosis in androgen-sensitive prostate cancer cells and inhibits tumour growth *in vivo*. J Cell Mol Med. (2015) 19:327–39. 10.1111/jcmm.12379 25444175 PMC4407608

[10] Zhu L Yuan H Jiang T Wang R Ma H Zhang S. Association of TLR2 and TLR4 polymorphisms with risk of cancer: a meta-analysis. PLoS ONE. (2013) 8:e82858. 10.1371/journal.pone.0082858 24376595 PMC3869723

[11] Basith S Manavalan B Yoo TH Kim SG Choi S. Roles of toll-like receptors in cancer: a double-edged sword for defense and offense. Arch Pharmacal Res. (2012) 35:1297–316. 10.1007/s12272-012-0802-7 22941474

[12] Takeda K Akira S. Toll-like receptors. Curr Protoc Immunol. (2015) 109:14–20. 10.1002/0471142735.im1412s109 25845562

[13] Paarnio K Tuomisto A Vayrynen SA Vayrynen JP Klintrup K Ohtonen P. Serum TLR2 and TLR4 levels in colorectal cancer and their association with systemic inflammatory markers, tumor characteristics, and disease outcome. APMIS Acta Pathol Microbiol Immunol Scand. (2019) 127:561–9. 10.1111/apm.12971 31132191

[14] Wang X Yu X Wang Q Lu Y Chen H. Expression and clinical significance of SATB1 and TLR4 in breast cancer. Oncol Lett. (2017) 14:3611–5. 10.3892/ol.2017.6571 28927120 PMC5587979

[15] Chow A Zhou W Liu L Fong MY Champer J Van Haute D. Macrophage immunomodulation by breast cancer-derived exosomes requires Toll-like receptor 2-mediated activation of NF-κB. Sci Rep. (2014) 4:5750. 10.1038/srep05750 25034888 PMC4102923

[16] Wang S Yao Y Rao C Zheng G Chen W. 25-HC decreases the sensitivity of human gastric cancer cells to 5-fluorouracil and promotes cells invasion via the TLR2/NF-κB signaling pathway. Int J Oncol. (2019) 54:966–80. 10.3892/ijo.2019.4684 30664194 PMC6365050

[17] Liu B Yan S Jia Y Ma J Wu S Xu Y. TLR2 promotes human intrahepatic cholangiocarcinoma cell migration and invasion by modulating NF-κB pathway-mediated inflammatory responses. FEBS J. (2016) 283:3839–50. 10.1111/febs.13894 27616304

[18] Liu YD Ji CB Li SB Yan F Gu QS Balic JJ. Toll-like receptor 2 stimulation promotes colorectal cancer cell growth via PI3K/Akt and NF-κB signaling pathways. Int Immunopharmacol. (2018) 59:375–83. 10.1016/j.intimp.2018.04.033 29689497

[19] Lu H Yang Y Gad E Inatsuka C Wenner CA Disis ML. TLR2 agonist PSK activates human NK cells and enhances the antitumor effect of HER2-targeted monoclonal antibody therapy. Clin Cancer Res. (2011) 17:6742–53. 10.1158/1078-0432.CCR-11-1142 21918170 PMC3206987

[20] Goldhirsch A Wood WC Coates AS Gelber RD Thurlimann B Senn HJ. Strategies for subtypes–dealing with the diversity of breast cancer: highlights of the St. Gallen international expert consensus on the primary therapy of early breast cancer 2011. Ann Oncol. (2011) 22:1736–47. 10.1093/annonc/mdr304 21709140 PMC3144634

[21] Singletary SE Allred C Ashley P Bassett LW Berry D Bland KI. Staging system for breast cancer: revisions for the 6th edition of the AJCC Cancer staging manual. Surg Clin N Am. (2003) 83:803–19. 10.1016/S0039-6109(03)00034-3 12875597

[22] Cardoso F Costa A Senkus E Aapro M Andre F Barrios CH 3rd ESO-ESMO international consensus guidelines for advanced breast cancer (ABC_3_). Ann Oncol. (2017) 28:16–33. 10.1093/annonc/mdw544 28177437 PMC5378224

[23] Ponde N Brandao M El-Hachem G Werbrouck E Piccart M. Treatment of advanced HER2-positive breast cancer: 2018 and beyond. Cancer Treat Rev. (2018) 67:10–20. 10.1016/j.ctrv.2018.04.016 29751334

[24] Kimbung S Loman N Hedenfalk I. Clinical and molecular complexity of breast cancer metastases. Semi Cancer Biol. (2015) 35:85–95. 10.1016/j.semcancer.2015.08.009 26319607

[25] Qiu N He Y Zhang S Hu X Chen M Li H. Cullin 7 is a predictor of poor prognosis in breast cancer patients and is involved in the proliferation and invasion of breast cancer cells by regulating the cell cycle and microtubule stability. Oncol Rep. (2018) 39:603–10. 10.3892/or.2017.6106 29207184

[26] Zhang N Zuo L Zheng H Li G Hu X. Increased expression of CD81 in breast cancer tissue is associated with reduced patient prognosis and increased cell migration and proliferation in MDA-MB-231 and MDA-MB-435S human breast cancer cell lines *in vitro*. Med Sci Monit. (2018) 24:5739–47. 10.12659/MSM.911612 30117494 PMC6109364

[27] Dai DN Li Y Chen B Du Y Li SB Lu SX. Elevated expression of CST1 promotes breast cancer progression and predicts a poor prognosis. J Mol Med. (2017) 95:873–86. 10.1007/s00109-017-1537-1 28523467 PMC5515997

[28] Bhatelia K Singh K Singh R. TLRs: linking inflammation and breast cancer. Cell Signal. (2014) 26:2350–7. 10.1016/j.cellsig.2014.07.035 25093807

[29] West AC Tang K Tye H Yu L Deng N Najdovska M. Identification of a TLR2-regulated gene signature associated with tumor cell growth in gastric cancer. Oncogene. (2017) 36:5134–44. 10.1038/onc.2017.121 28481875

[30] Echizen K Hirose O Maeda Y Oshima M. Inflammation in gastric cancer: interplay of the COX-2/prostaglandin E2 and Toll-like receptor/MyD88 pathways. Cancer Sci. (2016) 107:391–7. 10.1111/cas.12901 27079437 PMC4832872

[31] Qian J Luo F Yang J Liu J Liu R Wang L. TLR2 promotes glioma immune evasion by downregulating MHC class II molecules in microglia. Cancer Immunol Res. (2018) 6:1220–33. 10.1158/2326-6066.CIR-18-0020 30131377

[32] Xie W Wang Y Huang Y Yang H Wang J Hu Z. Toll-like receptor 2 mediates invasion via activating NF-κB in MDA-MB-231 breast cancer cells. Biochem Biophys Res Commun. (2009) 379:1027–32. 10.1016/j.bbrc.2009.01.009 19141294

[33] AlFakeeh A Brezden-Masley C. Overcoming endocrine resistance in hormone receptor-positive breast cancer. Curr Oncol. (2018) 25(Suppl. 1):S18–27. 10.3747/co.25.3752 29910644 PMC6001756

[34] De Angelis ML Francescangeli F Zeuner A. Breast cancer stem cells as drivers of tumor chemoresistance, dormancy and relapse: new challenges and therapeutic opportunities. Cancers. (2019) 11:1569. 10.3390/cancers11101569 31619007 PMC6826533

[35] Kim RS Avivar-Valderas A Estrada Y Bragado P Sosa MS Aguirre-Ghiso JA. Dormancy signatures and metastasis in estrogen receptor positive and negative breast cancer. PLoS ONE. (2012) 7:e35569. 10.1371/journal.pone.0035569 22530051 PMC3329481

[36] Adam AP George A Schewe D Bragado P Iglesias BV Ranganathan AC. Computational identification of a p38SAPK-regulated transcription factor network required for tumor cell quiescence. Cancer Res. (2009) 69:5664–72. 10.1158/0008-5472.CAN-08-3820 19584293 PMC2720524

[37] Almog N Ma L Raychowdhury R Schwager C Erber R Short S. Transcriptional switch of dormant tumors to fast-growing angiogenic phenotype. Cancer Res. (2009) 69:836–44. 10.1158/0008-5472.CAN-08-2590 19176381

[38] Keegan NM Gleeson JP Hennessy BT Morris PG. PI3K inhibition to overcome endocrine resistance in breast cancer. Expert Opin Investig Drugs. (2018) 27:1–15. 10.1080/13543784.2018.1417384 29252036

[39] Charmsaz S Hughes E Bane FT Tibbitts P McIlroy M Byrne C. S100beta as a serum marker in endocrine resistant breast cancer. BMC Med. (2017) 15:79. 10.1186/s12916-017-0836-2 28399921 PMC5389184

[40] Lee MH Koh D Na H Ka NL Kim S Kim HJ. MTA1 is a novel regulator of autophagy that induces tamoxifen resistance in breast cancer cells. Autophagy. (2018) 14:812–24. 10.1080/15548627.2017.1388476 29130361 PMC6070012

